# Does Hashimoto's Thyroiditis Increase the Risk of Cardiovascular Disease in Young Type 1 Diabetic Patients?

**DOI:** 10.3389/fendo.2020.00431

**Published:** 2020-07-24

**Authors:** Barbara Głowinska-Olszewska, Hanna Borysewicz-Sańczyk, Beata Sawicka, Bożenna Klonowska, Dorota Charemska, Beata Żelazowska-Rutkowska, Artur Bossowski

**Affiliations:** ^1^Department of Pediatrics, Endocrinology, Diabetology With Cardiology Division, Medical University of Białystok, Białystok, Poland; ^2^Department of Clinical Pediatrics, Faculty of Medical Sciences, Specialist Children's Hospital, University of Warmia and Mazury in Olsztyn, Olsztyn, Poland; ^3^Department of Paediatric Laboratory Diagnostics, Medical University of Białystok, Białystok, Poland

**Keywords:** diabetes type 1, Hashimoto's thyroiditis, cardiovascular risk, obesity, children, young adults

## Abstract

**Background:** Immunological and hormonal disorders have undoubted influence on the development of atherosclerotic process. Autoimmune diseases accompanying type 1 diabetes (T1D) may additionally accelerate atherosclerosis progression and increase the risk of cardiovascular events in the future. The influence of subclinical hypothyroidism on the cardiovascular system, in particular, has recently aroused great interest. The aim of our study was to assess intima-media thickness (cIMT) of common carotid arteries and the occurrence of classical atherosclerosis risk factors together with selected new biomarkers of cardiovascular diseases in young patients with type 1 diabetes mellitus coexisting with Hashimoto's disease (HD).

**Patients and Methods:** The study included 50 adolescents and young adults with T1D with mean age 17.1 ± 3 years, with mean diabetes duration of 10.5 ± 3.3 years, including 20 patients with diagnosed HD: T1D and HD(+), and 30 patients with no additional diseases: T1D and HD(–). Twenty-two healthy, age-matched volunteers formed control group (C). We analyzed mean HbA_1_c value from all years of disease, BMI, blood pressure, lipids, new biomarkers of atherosclerosis (hsCRP, adiponectin, myeloperoxidase, NT-proBNP peptide, vitamin D), and cIMT of common carotid arteries.

**Results:** In the group of patients with T1D and HD(+), significantly higher BMI was found: 23.3 ± 4.4 vs. 21.28 ± 2.9 in group HD(–) and 19.65 ± 2.4 kg/m^2^ in group C (*p* = 0.003), and higher waist circumference: 79 ± 10.9 vs. 75.10 ± 7.6 in group HD(–) vs. 69.0 ± 7.4 cm in group C (*p* < 0.001). The mean value of HbA_1_c was higher in group T1D and HD(+): 8.8% than in group HD(–): 8.1% (*p* = 0.04). Significantly higher concentration of hsCRP and lower vitamin D were observed in T1D and HD(+) in comparison to T1D and HD(–) and the control group. The IMT index in the HD(+) group was 0.46 ± 0.05 mm and was comparable to the HD(–) group but significantly higher than in healthy controls: 0.41 ± 0.03 mm (*P* < 0.05).

**Conclusions:** Young patients with type 1 diabetes mellitus and with coexisting Hashimoto's thyroiditis have a higher BMI, a higher waist circumference, and a higher HbA_1_c value, which altogether may cause faster development of macroangiopathy in the near future. Additional risk for cardiovascular disease may result from low vitamin D and increased hsCRP concentration in this group of patients. Coexistence of Hashimoto's thyroiditis did not significantly affect the cIMT value in the studied population.

## Introduction

Cardiovascular diseases (CVD) are the major chronic complications of type 1 diabetes mellitus (T1DM) and cause increased mortality (1). The estimated life expectancy is 14 years less for women and 17 years for men with childhood onset T1D (2). The risk of atherosclerosis development and early ischemic heart disease in T1DM patients is several times higher than in the general population (3). T1DM in children has been identified as a high-risk factor for premature development of CVD (4). Type 1 diabetes mellitus is also associated with a significantly higher prevalence of additional autoimmune diseases, including the incidence of Hashimoto's disease (HD) estimated at 3% to even 50% (5). The influence of subclinical hypothyroidism, including HD, on increased cardiovascular risk remains a current topic of research (6–9).

Over the last decade, atherosclerosis has been identified as an inflammatory disease involving pro-inflammatory cytokines that activate the expression of endothelial adhesion molecules, together with proteases and also other mediators (10). Inflammation concerns the formation of all stages of atherosclerotic lesions, including fatty acids, most commonly prevalent in children (11). The causes of inflammation in the vessel wall are not fully explained. According to one of the current hypotheses, atherosclerosis is an autoimmune disease. There is an increasing evidence provided by observing patients with diagnosed autoimmune diseases, especially lupus erythematosus, rheumatoid arthritis, or antiphospholipid syndrome. In the course of these diseases, atherosclerotic lesions develop rapidly and extensively, much faster and more often than in the general population. It seems that immunological dysregulation in the course of these diseases is crucial in accelerating the process of the autoimmune vascular damage. This suggests that the onset of atherosclerosis may be related to genetic predisposition to autoimmune diseases (12–14).

In the preclinical phase of the atherosclerosis process, great attention is paid to numerous “new biomarkers,” their usefulness in estimating the risk of cardiovascular disease, and explaining the complicated and still not fully understood pathogenesis of this disease (15). The last years confirmed the importance of high-sensitivity c-reactive protein (hsCRP) determination (16, 17). Clinical usefulness of many other biomarkers is discussed, of which oxidative stress markers, adiponectin, vitamin D, and atrial natriuretic peptide—NT-proBNP are documented both in basic and clinical studies (18, 19).

Non-invasive, ultrasonography-based studies performed among young people have shown the relationship between all known, traditional risk factors and abnormalities of blood vessel structure and function (20, 21). Recent studies have shown that T1DM already in children, adolescents, and young adults is associated with the greater carotid intima-media thickness (cIMT), the recognized marker of early structural atherosclerotic lesions (22). Nowadays, it is well-known that thickness of cIMT increases in patients with DMT1 as the disease progresses, and arterial parameters depend on the metabolic control but also on coexisting obesity, hypertension, and dyslipidemia (23–25).

Noteworthy, studies in patients who suffer from type 1 diabetes mellitus and additionally with coexisting autoimmune diseases regarding CVD risk factors, new biomarkers, and vascular status have not been conducted so far. Thus, the issue whether autoimmune diseases accompanying diabetes may further accelerate the progression of atherosclerosis and increase the risk of future cardiovascular events stays to be clarified. The current problem faced by young patients with all chronic diseases and especially DMT1 among them is not only life expectancy but also quality of life, which largely depends on the condition of the cardiovascular system.

Therefore, the purpose of the study was to evaluate cIMT (carotid intima-media thickness), classical cardiovascular risk factors, and selected new biomarkers of atherosclerosis in young patients with DMT1 with coexisting HD. We wanted to explain whether and how additional autoimmune disease in the course of type 1 diabetes mellitus in young people leads to accelerated development of atherosclerosis. We assumed that the new knowledge may help to create the appropriate therapeutic goals for these patients to minimize their cardiovascular risk and to understand better the mechanisms of atherosclerosis connected with thyroid autoimmunity.

## Patients

We recruited consecutive adolescents and also young adults diagnosed with type 1 diabetes remaining under the routine care of Children's Hospital and outpatient clinic in Olsztyn. The inclusion criteria for the study group were ages over 10 and under 26 and duration of illness at least 5 years. Criteria for exclusion from the study group were other types of diabetes, coexistence of other autoimmune disease (e.g., celiac disease), multiple autoimmune diseases in one patient, the occurrence of microvascular complications, previous recognition of hypertension or hyperlipidemia, and/or taking any additional drugs apart from insulin treatment and levothyroxine (from 0.5 to 2.0 mcg/kg body mass/day orally) if classified into the HD group.

Due to the diagnosis confirming the presence or absence of additional disease, patients were qualified to particular study groups: (1) group with diabetes mellitus type 1 and Hashimoto's thyroiditis (*n* = 20), and (2) group with DMT1 without additional accompanying disease (*n* = 30). Patients were qualified to particular groups on the basis of the results of periodic screening tests according to the guidelines presented by the Polish Diabetological Society and ESPE. The diagnosis of Hashimoto's thyroiditis was stated on the basis of standard criteria: elevated serum TSH level, decreased thyroid hormone (fT4) concentration always accompanied by elevated thyroid antibodies (aTPO and/or aTG) titer, and typical ultrasound. Among our patients, three persons were recognized with clinical hypothyroidism with decreased fT4. Two patients at recognition had elevated antibodies and had typical US picture indicating thyroid autoimmunity but with normal TSH and fT4, and the other 15 studied patients were diagnosed with subclinical hypothyroidism (elevated thyroid antibodies and TSH, fT4 within the norm with different degrees of thyroid gland involvement in ultrasonography). All included into the study were treated chronically with levothyroxine to keep TSH and fT4 within the normal range. To the Hashimoto group, we included patients with at least 1-year history of the additional diagnosis, with confirmed current euthyreosis (actual TSH and fT4 within normal range) status in laboratory tests.

**Figure 1:**
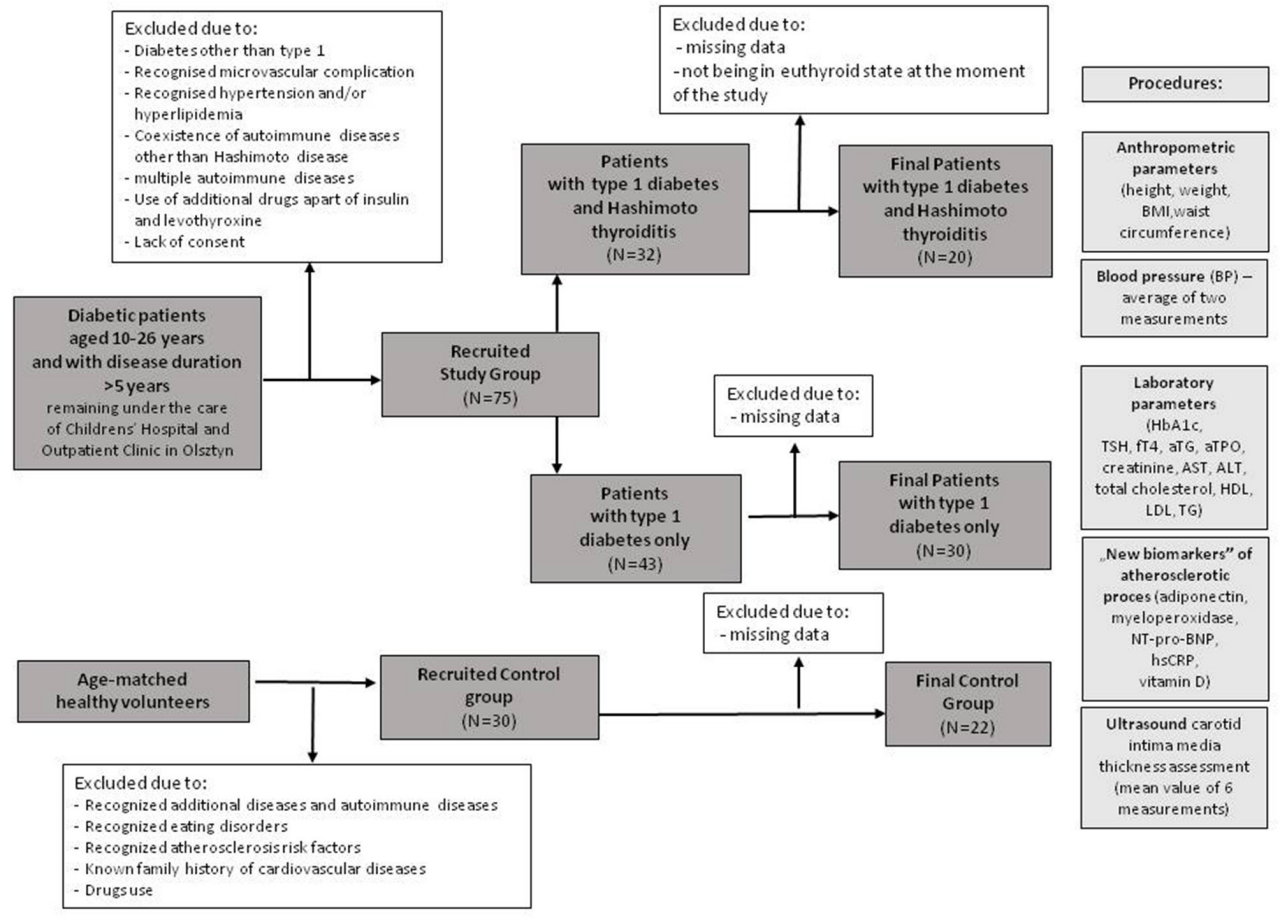
The flow chart of the selection for the study groups.

The reference group consisted of 22 healthy, age-matched volunteers. They were healthy, slim, normotensive students, young doctors, children of staff, and their friends. All of them had tests that excluded autoimmune and other diseases. People with mental disorders, including eating disorders (e.g., anorexia and bulimia), were not qualified for the study. The control group included individuals after exclusion of atherosclerosis risk factors (diabetes, hyperlipidemia, hypertension, and obesity) and without family history of cardiovascular diseases. Individuals in the reference group did not take any drugs. The recruitment process is presented in the flow chart (Figure 1).

The study protocol was approved by the Bioethics Committee of the Warmia and Mazury Chamber of Physicians and Dentists in Olsztyn, Poland. In each case of a juvenile patient (below 16 years), his or her parents'/guardians', and in the case of persons aged 16 years and over, their personal, informed written consent forms were obtained—in order to participate in the study.

## Methods

All patients had to undergo physical examination. Their height and weight were measured in a standard way by using a Harpenden stadiometer and a digital scale. Then their body mass index (BMI) was counted on the base of a standard formula. In order to adjust for age and sex, the BMI standard deviation score (BMI-SDS) was calculated and assessed using age- and sex-specific BMI growth charts according to a local Polish OLAF study (26). Patients were divided as normal weight, overweight, or obese depending on the BMI-SDS. Waist circumference was measured with clinic centimeter and converted to waist-SDS. There were two measurements of the systolic blood pressure (SBP) and the diastolic blood pressure (DBP) at the right arm, each one after a 10-min rest with the use of calibrated sphygmomanometer of the proper cuff size, and the readings were averaged.

### Laboratory Analyses

For laboratory tests, venous blood was collected after 8–12 h of fasting. Eight milliliters of blood was collected and then centrifuged for 10 min at 2,000 turns per minute. Several of the variables (HbA_1_c, lipids, vitamin D, hormones, and thyroid antibodies) were performed on an ongoing basis in the hospital laboratory using standard methods. HbA_1_c was evaluated in two ways: the last value in time when the blood sample was taken, and the mean value from the total time of disease duration. The remaining material (serum) was stored at a temperature of −80°C until the determination. Adiponectin (Adp), natriuretic peptide (NT-proBNP), and myeloperoxidase (MPO) markers were analyzed immunoenzymatically using ELISA kits that are commercially accessible (Parameter Human Immunoassays, R&D Systems, Inc., Minneapolis, USA). hsCRP was determined owing to the immunoturbidimetric method [Tina-quant hsCRP (Latex) HS, Roche; Hitachi 912, La Roche, Japan]. Serum levels of free thyroxine (fT4) and TSH were calculated on electrochemiluminescence, ECLIA, with Cobas E411 analyzer (Roche Diagnostics). The range of normal values for fT4 was between 1.1 and 1.7 ng/dL, and that for TSH was between 0.28 and 4.3 (μIU/l). Anti-TPO and anti-TG antibodies were measured in all samples using ECLIA with Modular Analytics E170 analyzer (Roche Diagnostics). The positive values for antibodies were >34 IU/mL for anti-TPO-Abs and >115 IU/mL for anti-TG-Abs.

#### Ultrasound Measurements

The procedure of ultrasound measurements was conducted in the timeslot between 8:00 and 10:00 a.m., and after a fasting period from 8 to 12 h. Measuring of intima-media thickness (IMT) in the right and left common carotid arteries was conducted as described in previous methodology, with our own modification (27, 28). Measuring covered end-diastolic (minimum diameter) IMT of the far walls (the distance between the leading edge of the first echogenic line and the leading edge of the second echogenic line) within a distance larger than 1 cm from the bifurcation. The mean value of six measurements (three from the left and three from the right carotid artery) was included in the analyses. The representative images for two groups (DMT1 and controls) with graphic scheme for IMT are presented in the Supplementary Figures 1–3.

### Statistical Analysis

The statistical analysis was performed using Statistica 12.0 (Stat Soft, USA). All the continuous variables were tested for normal distribution by the Kolmogorov-Smirnov, with Lilliefors correction and Shapiro-Wilk tests. For variables meeting the criteria of normal distribution, the Student's *t*-test was used when comparing two variables. In the analysis of more than two groups, the analysis of variance was used with Tukey's *post-hoc* RIR test for unequal numbers. The results are presented as mean ± standard deviation *(SD)*. Non-parametric tests were used for variables not meeting the criteria of normal distribution. Mann-Whitney U non-parametric test was applied to compare quantitative variables. In the case of comparisons for more than two groups, the ANOVA rang Kruskal-Wallis test and the median test with *post-hoc* tests of multiple comparisons were used for all samples. Results are shown as median (Me) and interquartile range or mean and SD. We performed a *post-hoc* sample size calculation basing on our outcome to achieve a power of 1-β = 0.70–0.80 for the ANOVA Kruskal-Wallis test at level α = 0.05. Under these assumptions, an amount of a minimum 20 participants per group is required.

The analysis of correlations was performed using the Spearman test with the determination of the rank-order (rho) correlation coefficient. In order to detect independent determinants of IMT, multivariate regression analysis was performed. Only variables for which the *p*-value in a univariate analysis was < 0.05 were included in this model. All comparisons were adjusted to age, gender, BMI, and blood pressure values. Statistically significant results were found at the level of *P* < 0.05.

## Results

We recruited a total of 50 patients with diabetes type 1 (20, 40% males), aged mean 17.1 + 3 years, with mean diabetes duration of 10.3 + 3.1 years, mean HbA_1_c from the whole disease at 8.4 + 1.3%, and HbA_1_c at the time of the analysis at 8.7 + 1.2%. Ninety-two percent of the patients were treated with continuous subcutaneous insulin infusion (CSII). Twenty patients (7, 35% males) were diagnosed with HD [T1D HD(+)]. Thirty patients had T1D without any other additional diseases [T1D HD(–)]. Studied groups were similar in mean age, diabetes duration, metabolic control, and daily insulin requirement. Body mass was higher in the HD(+) group (*P* = 0.006). TSH level was significantly (*P* = 0.002), and fT4 insignificantly higher in the T1D HD(+) group, although all values stayed within the normal range. The control group consisted of 22 (9, 41% males), age/gender-matched healthy volunteers. The general characteristic of the study groups is shown in Table 1.

**Table 1:** General characteristics of the study groups.

	**DMT1** **total** ***N* = 50**	**DMT1 group** **with Hashimoto's** **thyroiditis** ***N* = 20**	**DMT1 group** **without Hashimoto's** **thyroiditis ** *N* **=** **30**	**Control group** ***N* = 22**	***P*-values**
Age (years)	17.1 ± 3	17.6 ± 3.1	16.8 ± 3.0	16.5 ± 5.0	0.62^**^
Gender (M/F) [*n* (%)]	20 (40%)/30 (60%)	7 (35%)/13 (65%)	13 (45%)/17 (57%)	9 (41%)/13 (59%)	
Diabetes duration (years)	10.3 ± 3.1	10.8 ± 3.5	10.0 ± 2.8		0.33^*^
Age of onset (years)	6.8 ± 3.6	6.8 ± 3.4	6.8 ± 3.9		0.90^*^
Body mass (kg)	63.4 ± 14.5	68.3 ± 15.3^a^	60.3 ± 13.3	54.0 ± 13.8	0.006^**^
Height (cm)	170.6 ± 11	170.8 ± 11.6	170.5 ± 11.2	164.4 ± 13.0	0.13^**^
HbA_1_c mean (total disease duration time) (%)	8.4 ± 1.3	8.8 ± 1.4	8.1 ± 1.1		0.06^*^
HbA_1_c last (%)	8.7 ± 1.2	9.0 ± 1.2	8.6 ± 0.4	5.4 ± 0.3	0.17^*^
Daily insulin requirement (UI/kg/24 h)	0.8 ± 0.18	0.8 ± 0.17	0.8 ± 0.16		0.65^*^
Remission period (months)	7.9 ± 7.6	6.6 ± 7.6	8.8 ± 7.6		0.35^*^
TSH uIU/L	2.9 ± 0.9	3.5 ± 0.6^a^^,^ ^b^	2.4 ± 0.8	2.33 ± 1.24	0.002^**^
fT4	1.33 ± 0.28	1.37 ± 0.19	1.31 ± 0.33	1.32 ± 0.24	0.61^**^
aTPO IU/ml	94 ± 185	209 ± 255^a^^,^ ^b^	17 ± 16	18 ± 10	<0.005^**^
aTG IU/ml	432 ± 987	1030 ± 1370^a^^,^ ^b^	34 ± 68	53 ± 36	<0.005^**^
Creatinine mg/dl	0.75 ± 0.16	0.76 ± 0.16	0.75 ± 0.16	0.76 ± 0.2	0.92^**^
AST U/L	25.1 ± 18	31.6 ± 26.4	21.6 ± 7.4	23.5 ± 6.5	0.07^**^
ALT U/L	28 ± 10	29.4 ± 11.3	27.1 ± 10.0	24.6 ± 7.1	0.27^**^

* p-values in t-student test [difference between Hashimoto (+) and Hashimoto (–) patients].

** p-values in ANOVA variance test (differences between both diabetic and control groups).

a p < 0.05—compared to the control group.

b *p < 0.05—compared to the diabetes and Hashimoto (–) group in post-hoc tests. The data are presented as mean ± SD*.

**Figure 2:**
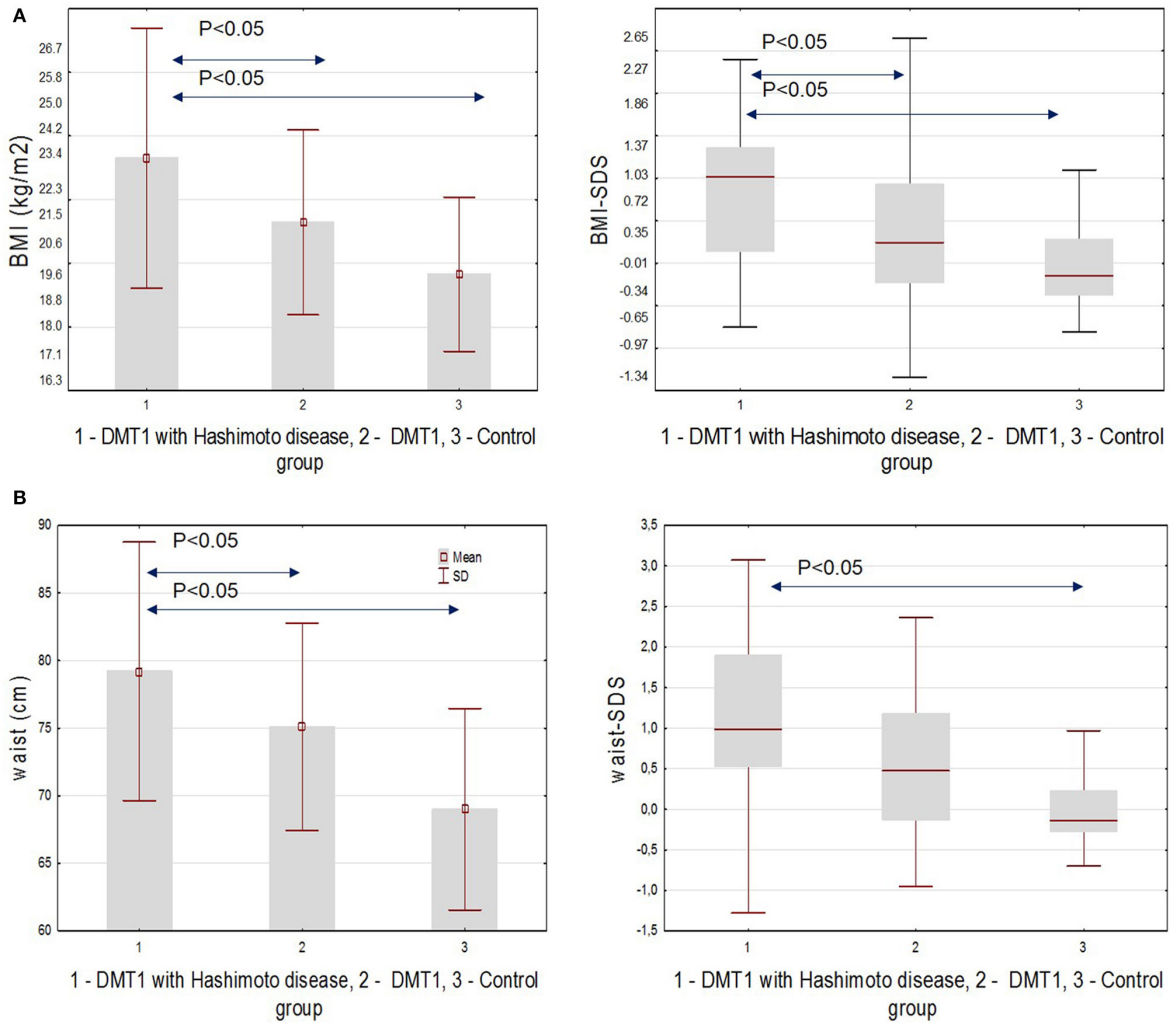
**(A)** BMI and SDS-BMI in the studied groups. **(B)** Waist circumference and waist-SDS in the studied groups.

First, we analyzed classical risk factors of cardiovascular diseases. We found significantly higher BMI and SDS–BMI in patients with T1D and HD(+) compared to T1D and HD(–) and to control groups (*p* = 0.003, *p* = 0.010, respectively). Nine patients (45%) from the HD(+) group were found to be overweight or obese. Waist circumference was higher in the HD(+) group compared to both the remaining groups (*p* < 0.001), and waist-SDS was significantly higher in comparison with the control group (*p* = 0.002). SBP was higher among both HD(+) and HD(–) compared to controls (*p* < 0.001), and DBP was the highest in T1D and HD(+) (*p* = 0.008). Within lipid parameters, we found significant differences in the triglycerides level, with the highest values in the T1D and HD(+) groups (*p* = 0.005 in comparison to controls). The HbA_1_c value, averaged from the whole disease period, was higher in HD(+) (*p* = 0.04) and comparable with the HD(–) group when the last value from the time of the current analysis was considered (Table 2, Figure 2).

**Table 2:** Comparison of clinical parameters, lipid levels, metabolic control, and analysis of the concentration of “new biomarkers” of the atherosclerotic process between study groups.

	**DMT1 group with Hashimoto's thyroiditis** ***N*** **= 20**	**DMT1 group** ***N* = 30**	**Control group** ***N* = 22**	*P*-values^*^
BMI (kg/m^2^)	23.3 ± 4.4^a^^,^ ^b^	21.28 ± 2.9	19.65 ± 2.4	0.003
BMI-SDS	1.015 (0.14–1.36)^a^^,^ ^b^	0.24 (−0.2–0.93)	−0.1 (−0.3–0.28)	0.010
Waist (cm)	79 ± 10.9^a^^,^ ^b^	75.1 ± 7.6	69.0 ± 7.4	<0.001
Waist-SDS	0.98 (0.52–1.9)^a^	0.47 (−0.13–1.18)	−0.14 (−0.27–0.23)	0.002
SBP (mmHg)	125 ± 15^a^	121.5 ± 11^a^	109 ± 9	<0.001
DBP (mmHg)	74 ± 8^a^	71.1 ± 6	69 ± 5	0.008
Total cholesterol (mg/dl)	180 ± 30	176 ± 25	164 ± 29	0.160
LDL (mg/dl)	96 ± 31	101 ± 28	89 ± 28	0.100
HDL(mg/dl)	57 ± 10	57 ± 11	59 ± 11	0.150
TG (mg/dl)	108 ± 354^a^	82 ± 27	75 ± 39	0.005
HbA_1_c mean %	8.8 ± 1.4	8.1 ± 1.1	–	0.040^**^
HbA_1_c last %	9.0 ± 1.2^a^	8.6 ± 1.1^a^	5.4 ± 0.2	<0.001
Adiponectin (ng/ml)	8764.3 (6659–14616)	7704.6 (4816–10231)	9746.6 (4933–11333)	0.650
Myeloperoxidase (ng/ml)	184.6 (120–325)^a^	200.8 (95–281)^a^	96.8 (72–139)	0.012
NTproBNP (pg/ml)	29.5 (17.8–40.0)	23.4 (15.2–43.8)	28.9 (17–37)	0.080
hsCRP (mg/L)	0.98 (0.4–2.49)^a, b^	0.36 (0.23–0.69)	0.2 (0.1–0.31)	<0.001
Vitamin D (ng/ml)	17.9 ± 7.9^a^	18.5 ± 8.1^a^	25.4 ± 5.7	<0.001

The data are presented as mean ± SD or median (interquartile range).

* ANOVA Kruskal-Wallis test.

** t-student test.

a p < 0.05—compared to the control group.

b *p < 0.05—compared to the diabetes group in post-hoc tests*.

**Figure 3:**
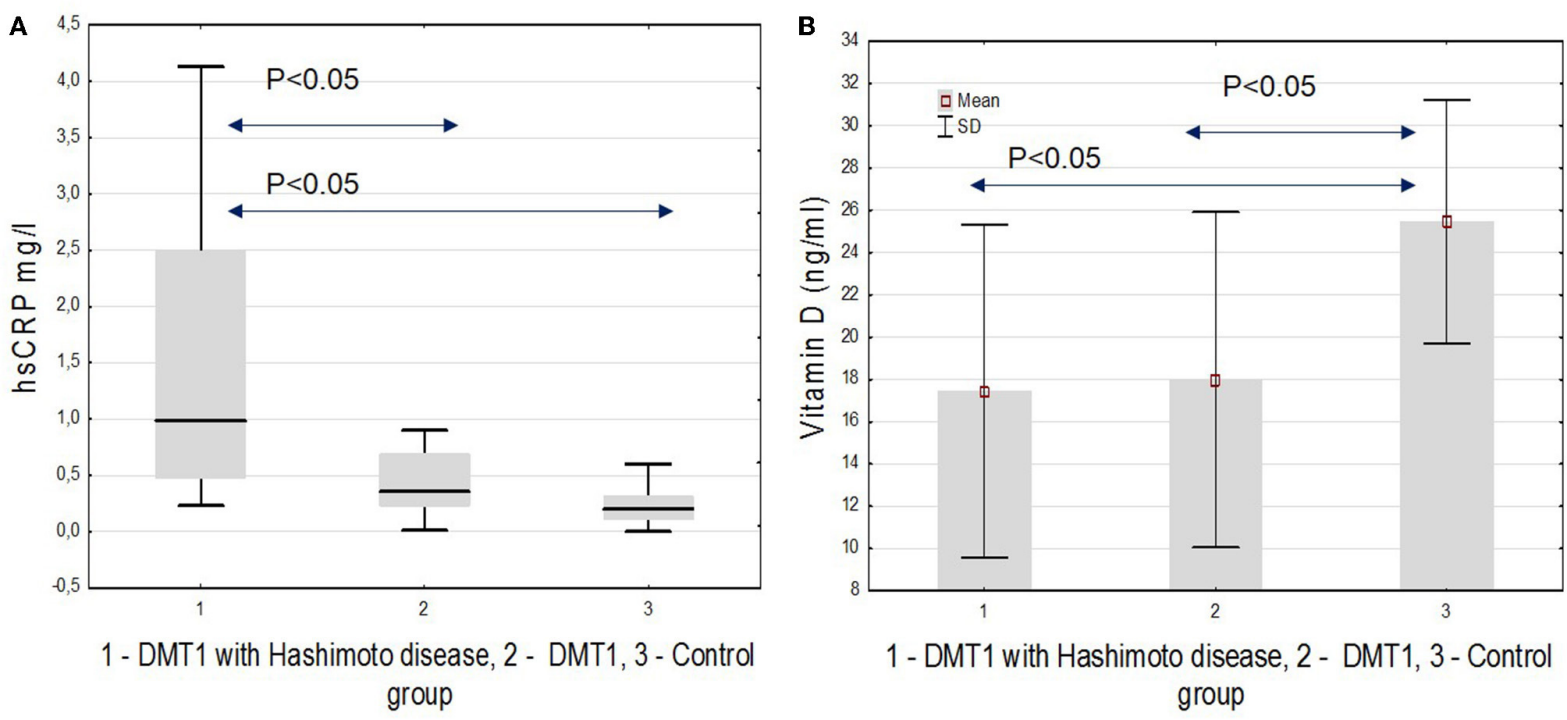
**(A)** hsCRP. **(B)** Vitamin D in the studied groups.

Next, we analyzed the differences in new biomarkers of the atherosclerotic process. We showed significant differences in the myeloperoxidase level that was higher in both diabetic groups in comparison with controls (*p* = 0.012), hsCRP, higher in T1D and HD(+) compared to T1D and HD(–) (Figure 3A), and to the control groups (*p* < 0.001), as well as in the vitamin D level, which we found lower in both diabetic groups compared to healthy ones (*p* < 0.001) (Figure 3B, Table 2).

**Figure 4:**
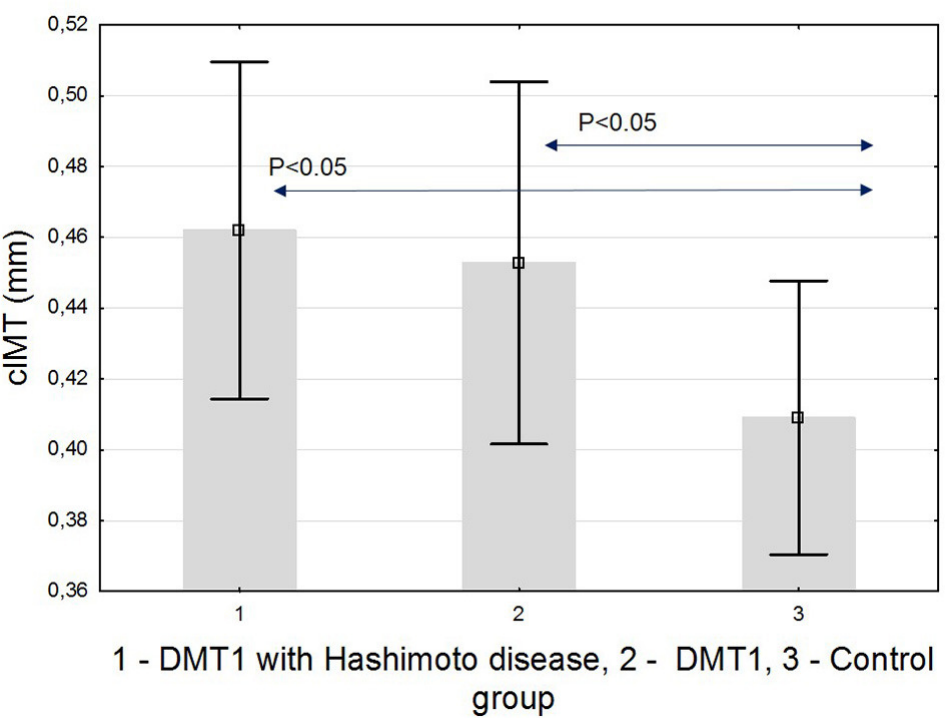
cIMT in the studied groups.

**Figure 5:**
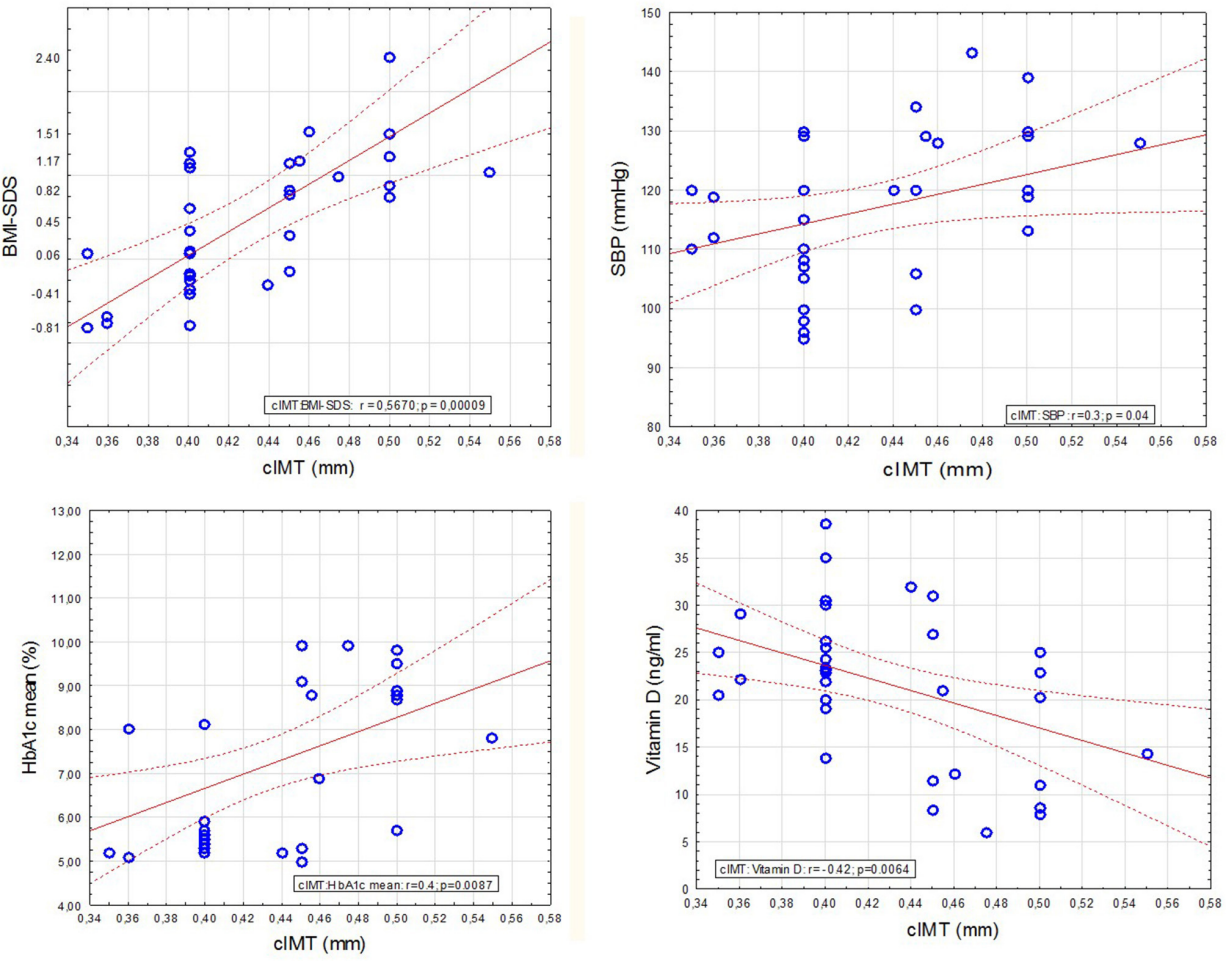
Analysis of IMT correlation with selected variables in patients with diabetes type 1 and Hashimoto's thyroiditis.

Finally, we analyzed the IMT value. The thickness of the intima-media of the common carotid arteries was significantly higher in both diabetic groups: 0.46 ± 0.05 mm in T1D with HD(+), 0.45 ± 0.04 mm in T1D with HD(–) compared to the control group: 0.41 ± 0.03 mm (both *p* < 0.05) (Figure 4). IMT correlated significantly positively with BMI-SDS, SBP, and mean HbA_1_c, and negatively with vitamin D (Figure 5). In Table 3, we present other results of the correlation analysis between IMT and studied classical risk factors and new biomarkers of cardiovascular disease in the group of HD(+) patients. In the multivariate regression model regarding this group, IMT was associated significantly with SDS-BMI and vitamin D level (*R*^2^ = 0.48, B = 0.18, *p* < 0.04).

**Table 3:** Correlation analysis between cIMT and other studied variables in DMT1 with Hashimoto's thyroiditis group.

	**cIMT**
Age	Rho = 0.280, *P* = 0.075
Diabetes duration	Rho = 0.030, *P* = 0.860
BMI	Rho = 0.580, *P* < 0.001
BMI-SDS	Rho = 0.560, *P* < 0.001
Waist SDS	Rho = 0.490, *P* < 0.001
SBP	Rho = 0.300, *P* = 0.04
DBP	Rho = 0.370, *P* = 0.013
Total cholesterol	Rho = 0.019, *P* = 0.900
LDL-cholesterol	Rho = 0.001, *P* = 0.100
HDL-cholesterol	Rho = 0.080, *P* = 0.570
Triglicerides	Rho = 0.140, *P* = 0.340
HbA_1_c mean	Rho = 0.400, *P* = 0.009
Adiponectin	Rho = −0.040, *P* = 0.810
Myeloperoxidase	Rho = 0.170, *P* = 0.350
NT proBNP	Rho = 0.230, *P* = 0.170
hsCRP	Rho = 0.280, *P* = 0.075
Vitamin D	Rho = −0.420, *P* = 0.006

## Discussion

The crucial finding of our study is that young patients (teenagers and young adults) with T1D and coexisting additional Hashimoto's disease HD(+) have much more unfavorable profile of classical cardiovascular risks factors compared to T1D peers without any additional disease. We found higher body mass, waist circumference, blood pressure values, and triglycerides concentration, as well as poorer metabolic control, evaluated as mean glycated hemoglobin from the whole disease period and just the last value. In the present study, we also confirmed that patients with recognized HD had higher concentration of hsCRP compared not only to the healthy group but also to the T1D HD(–) group. Moreover, the myeloperoxidase level was higher, whereas the vitamin D concentration was lower in both groups of diabetic patients. However, the cIMT value was comparable between HD(+) and HD(–) T1D patients, and in both groups, considerably higher than in the control group.

In the current study, we chose to evaluate the population of teenagers and young adults with T1D. To the best of our knowledge, they represent the rarely studied population of diabetic patients. CV risk factors are quite commonly studied in diabetic children groups or in adult ones when clinical complications and apparent macroangiopathy have already appeared. Our study provides the unique possibility to get the knowledge on CV risk factors status in T1D patients being almost or already young adults, with quite a long time of diabetes duration (at least 5 years), yet without confirmed cardiovascular complications. As the main target, however, we decided to investigate the group with coexistence of autoimmune hypothyroidism. Additional autoimmune diseases, among them mainly thyroid autoimmunopathies, are frequent comorbidities of T1D. Their prevalence increases with diabetes duration (29), and generally, the frequency of additional autoimmune diseases is increasing in the last decades (5). Just a few studies published so far have presented the data of single cardiovascular risk factors, specifically dyslipidemia, in pediatric diabetic patients with coexisting thyroid autoimmunity (30, 31).

Multiple studies in patients with subclinical hypothyroidism have shown the association with cardiovascular abnormalities, like impaired endothelial function, increased IMT, left ventricular dysfunction, heart failure, coronary artery disease, and cardiovascular death. Many of these studies proved the substantial contribution of dyslipidemia, hypertension, obesity, insulin resistance, and metabolic syndrome in these complications (7, 32). The recently published meta-analyses on early atherosclerosis in SH patients showed that severity of thyroid hormones disturbance is closely associated with the degree of arteries' function and structure, but other factors, like additional diseases, could not be ruled out (32, 33). The scientific research on the relevance of low-normal thyroid function on components of the metabolic syndrome (MS) shows that the state is significantly associated with all components of MS (34). Several studies in obese and also in non-obese individuals, with thyroid function at normal range, presented the results of association between elevated thyroid antibodies with insulin resistance and hsCRP (16, 35). Low-normal thyroid function may be implicated into atherosclerosis development via connections with insulin resistance and metabolic syndrome (36). All our patients with HD(+) were in laboratory euthyroid state, and to make this group more homogeneous, all included subjects were treated with supplemental dose of levothyroxine. Notwithstanding, we observed significant differences in the TSH level between the HD(+) and HD(–) groups, which may be one of the explanations of observed differences in CV risk factors intensity.

Studies considering early atherosclerosis risk in childhood thyroid autoimmunity are scarce, and the results are inconsistent. In euthyroid girls newly diagnosed with HD, increased total cholesterol and hsCRP levels were found, like in our study, but also increased cIMT and no differences in BMI contrary to our results (37, 38). In another study conducted on a large group of Spanish children, higher levels of thyrotropin were found in obese young patients. The difference between obese and normal weight may be related to higher incidence of thyroid autoimmunity in the overweight patients (39). Isolated increased TSH was found to be common in other obese pediatric population, without significant relationship to autoimmune status (40). Recently published meta-analysis clearly indicated that obesity was independently significantly associated with hypothyroidism, recognition of HD, and thyroid antibodies (41).

Our current analyses proved that more altered parameters associated with CV risk were found in the group of T1D with HD. This group had the highest BMI, expressed also as SDS-BMI, and waist circumference, which is the key index in insulin resistance recognition in clinical settings. Almost half of the group fulfilled criteria for overweight or obesity. Ciccone et al. presented the results of the study in women with Hashimoto's thyroiditis, where they found that IMT is increased only in obese and overweight patients. This correlation between Hashimoto's thyroiditis and IMT seemed to be independent of TSH and thyroid hormone values. They conclude that HD represents a marker of atherosclerosis development when combined to adiposity (42).

The increasing prevalence of obesity worldwide is parallel to increasing numbers of not only cardiovascular diseases or cancers but autoimmune conditions as well. Obesity is regarded as a chronic low-grade inflammation process, where many inflammatory markers and cytokines are overproduced and over-activated. This process may result in increased pathogenic processes leading up to increased outbreak of type 1 diabetes, higher numbers of autoimmune thyroiditis, and cardiovascular disease in the future. Obesity seems to be a core environmental contributing factor to the onset and development of autoimmune diseases (43).

The excess in body weight has become an urgent problem among patients with T1D. It is reported that T1D is being recognized with higher SDS-BMI nowadays, and there is a trend for increasing BMI with diabetes duration (44). As many as 30% of young diabetic patients are overweight or obese (45, 46). Very alarming data come from recent DCCT/EDIC published studies. This is a crucial, longitudinal observation for T1D intensive insulin treatment implementation. The results of the study proved, firstly, that intensive insulin treatment regimens result in improvement in metabolic control and significant reduction in IMT and all vascular complications rates, CVD, and myocardial infarctions among them (47). Recent observation, however, found that these subjects from the intensive group, who experienced excessive weight gain, had increased IMT and total CVD event after 15 years observation, comparing to the group treated conventionally, thus with poorer metabolic control. Weight gain in long-term observation seems to nullify the success of intensive insulin therapy and improved metabolic control (48). The issue whether evolving, obesity-connected autoimmunity in T1D patients additionally exacerbates the risk of early CVD remains unexplained.

In our group of T1D and HD(+) patients, we also confirmed elevated triglycerides level. The amount of already published data proved that thyroid dysfunction and autoimmune process, even in young population, is connected with impaired lipid metabolism. Severe atherogenic dyslipidemia may occur in overt hypothyroidism, while in euthyroid AIT patients, the alterations are discrete. The lack of thyroid hormones is related to reduced clearance of TG-rich particles. Hypertriglyceridemia has been associated with the increased production of small, dense LDL (49). Long-term consequences of childhood AITD-associated dyslipidemia remain unknown, but the short–term data reveal improvement in lipid profile with L-thyroxine treatment [reviewed in (50)]. Atherogenic dyslipidemia is a huge, recognized but undertreated and pending problem among young people with T1D (46, 51, 52).

In our study, we decided not to limit analyses only to traditional risk factors but to investigate selected new biomarkers of atherosclerosis as well. The relevance of hsCRP as a new and independent atherosclerosis biomarker, associated mainly with obesity and low-grade inflammatory state, is established (10, 17). A correlation between cIMT and adiponectin, leptin, and high C-reactive protein (hsCRP) has been demonstrated in obese children (53, 54). Here in our study, we proved higher hsCRP level in patients with HD(+). Our data showed decreased concentration of vitamin D in both groups of diabetic patients. Some studies reported correlation between the deficiency of vitamin D and the risk of autoimmune disease (55, 56). Several observations found decreased vitamin D levels in obese and T1D patients (57, 58). What is more interesting, supplementation with vitamin D was associated with an improvement in peripheral vascular function in diabetic children (57, 58). The discussion whether supplementation with vitamin D may be preventive in general or selected population in CVD prevention is open (59).

Both our presented diabetic groups had significantly higher cIMT compared to the healthy group. The difference between the HD(+) and HD(–) groups was not apparent, although many differences in cardiovascular risk intensity were noticed and discussed above that indicated that HD(+) should be at higher risk. In our patients, IMT was correlated with BMI, SBP, and HbA_1_c. These results are in line with already published data considering young diabetic patients (24, 60). Both our diabetic groups had poor metabolic control. The mean disease value was far from recommendations. Unfortunately, this is a well-recognized clinical problem that pediatric and young population is very problematic in keeping proper metabolic control even with modern technologies (61). We think that it is possible that chronic hyperglycemia at this teenager age remains the main contributor to IMT, like some other authors found (62). However, contrary to ours and the studies discussed above, the results of SEARCH CVD Study clearly stated that CV risk factors burden increased gradually in young people with T1D, BMI was a major risk modifiable factor that was predicting carotid IMT, and HbA_1_c alone could not explain the value of IMT (24). So far, additional autoimmune processes were not included into such analyses among T1D patients.

cIMT was proved to be increased in clinically overt hypothyroidism, and the decrease was noticed after thyroxin treatment (63). However, there are also some reports that prove the increased cIMT in euthyroid, non-diabetic state but connected with autoimmune thyroid condition (64). The issue of significance of pharmacological treatment among patients with SH remains open. In some studies, thyroxin replacement was related to significant reduction in carotid IMT, and improving lipid profile (65, 66).

The issue whether HD as an autoimmune condition may be responsible for autoimmune, inflammation-based endothelial dysfunction itself remains to be elucidated. However, some studies demonstrated these early vessels impairment in HD patients to be independent from other risk factors for CVD (67). An increased ongoing inflammatory status might contribute to increased insulin resistance in both obese and non-obese AIT patients even with euthyroidism (35). It should be established whether HD is an independent cardiovascular risk factor. The possible pathogenic mechanism of the connection between HD, type 1 diabetes, obesity, and early atherosclerosis remains unclear. However, several hypotheses can be discussed. In patients without T1D, it was proved that IMT is related to hormone levels, even when their values remain within the normal range. In our group, we confirmed that the T1D HD(+) patients had higher TSH level despite the pharmacological treatment, and the level of thyroid antibodies remained high, indicating ongoing autoimmune process. The lack of difference in IMT values between HD(+) and HD(–) patients can be explained by probably the strongest influence of poor metabolic control in all diabetic patients. Metabolic control, expressed as the HbA_1_c level, is known to be the strongest cardiovascular risk factor in children with T1D. Recently published DCCT/EDIC study population data clearly revealed that HbA_1_c is associated with numerous traditional CVD risk factors, and that this association cannot alone be an explanation of its effect on the CVD risk. It is concluded that aggressive management of traditional non-glycemic CVD risk factors is indicated in all T1D patients and, together with excellent metabolic control, remains the primary objective (68). However, it cannot be entirely excluded that autoimmune thyroiditis may itself be causing inflammation of autoimmune origin that keeps atherosclerosis process accelerated in the long run. Randomized, controlled, and longitudinal studies on larger patient groups with T1D and HD(+) are needed to prove the benefits of additional early levothyroxine replacement on reducing the CVD risk in young patients with diabetes type 1, additionally to continuous efforts for improving metabolic control. Long-term cardiovascular consequences of T1D in today's young patients, affected additionally by autoimmune hypothyroidism, remain unknown due to lack of longitudinal prospective studies.

## Limitations of the Study

We are aware that there are certain limitations of our study implicating a careful interpretation of the study results. The main limitation of our study is the small sample size of the population and the small number of patients included into every studied group. Moreover, all patients came from the same one center. However, the sample size calculation allowed us to carry out the designed study. Another limitation is that we did not perform screening tests for other autoimmunities, except for celiac disease. There are no screening recommendations because of the rare occurrence among diabetic type 1 patients. Additional diseases, other than thyroid and celiac, are diagnosed on the basis of clinical presentation firstly. We did not recruit into the study group these patients with recognized autoimmune disease other than Hashimoto's thyroiditis, so we cannot exclude that among our patients, there might have been any additional subclinical autoimmune processes.

## Conclusions

Young patients suffering from type 1 diabetes mellitus and with coexisting Hashimoto's thyroiditis have a higher BMI, a higher waist circumference, and a higher HbA_1_c value, which altogether may cause faster development of macroangiopathy in the near future. Additional risk for cardiovascular disease may result from low vitamin D and increased hsCRP concentration in this group of patients. Coexistence of Hashimoto's thyroiditis did not significantly affect the cIMT value in the studied population. Explaining whether and how additional autoimmune diseases in the course of type 1 diabetes mellitus in young people lead to accelerated development of atherosclerosis can help not only to create the right therapeutic goals for these patients to minimize their cardiovascular risk but also may be the next step in understanding the autoimmune mechanisms of atherosclerosis.

## Data Availability Statement

The raw data supporting the conclusions of this article will be made available by the authors, without undue reservation.

## Ethics Statement

The studies involving human participants were reviewed and approved by Bioethics Committee of the Warmia and Mazury Chamber of Physicians and Dentists in Olsztyn, Poland. Written informed consent to participate in this study was provided by the participants' legal guardian/next of kin.

## Author Contributions

BG-O designed the study, performed the statistical analysis, and drafted and wrote the manuscript. HB-S and BS analyzed the data, participated in the study conception, and designed and contributed to a great extent to the discussion. BK and DC participated in patients' recruitment, collecting the data, and analyses. BŻ-R performed the laboratory analyses of new biomarkers and analyzed them. AB was involved in the design, conception, analysis, and revision of the manuscript. All authors contributed in discussions and read and approved the final version of the manuscript.

## Conflict of Interest

The authors declare that the research was conducted in the absence of any commercial or financial relationships that could be construed as a potential conflict of interest.

## Supplementary Material

Supplementary Figure 1 The graphic scheme of IMT measurement.

Supplementary Figure 2 IMT ultrasonography representative image for diabetic patient.

Supplementary Figure 3 IMT ultrasonography representative image for healthy control.
