# Supplementary figures and images for: Does Hashimoto's Thyroiditis Increase the Risk of Cardiovascular Disease in Young Type 1 Diabetic Patients?
Source: Front Endocrinol (Lausanne). 2020 Jul 24;11:431. doi: 10.3389/fendo.2020.00431 (PMC7393727; doi:10.3389/fendo.2020.00431)

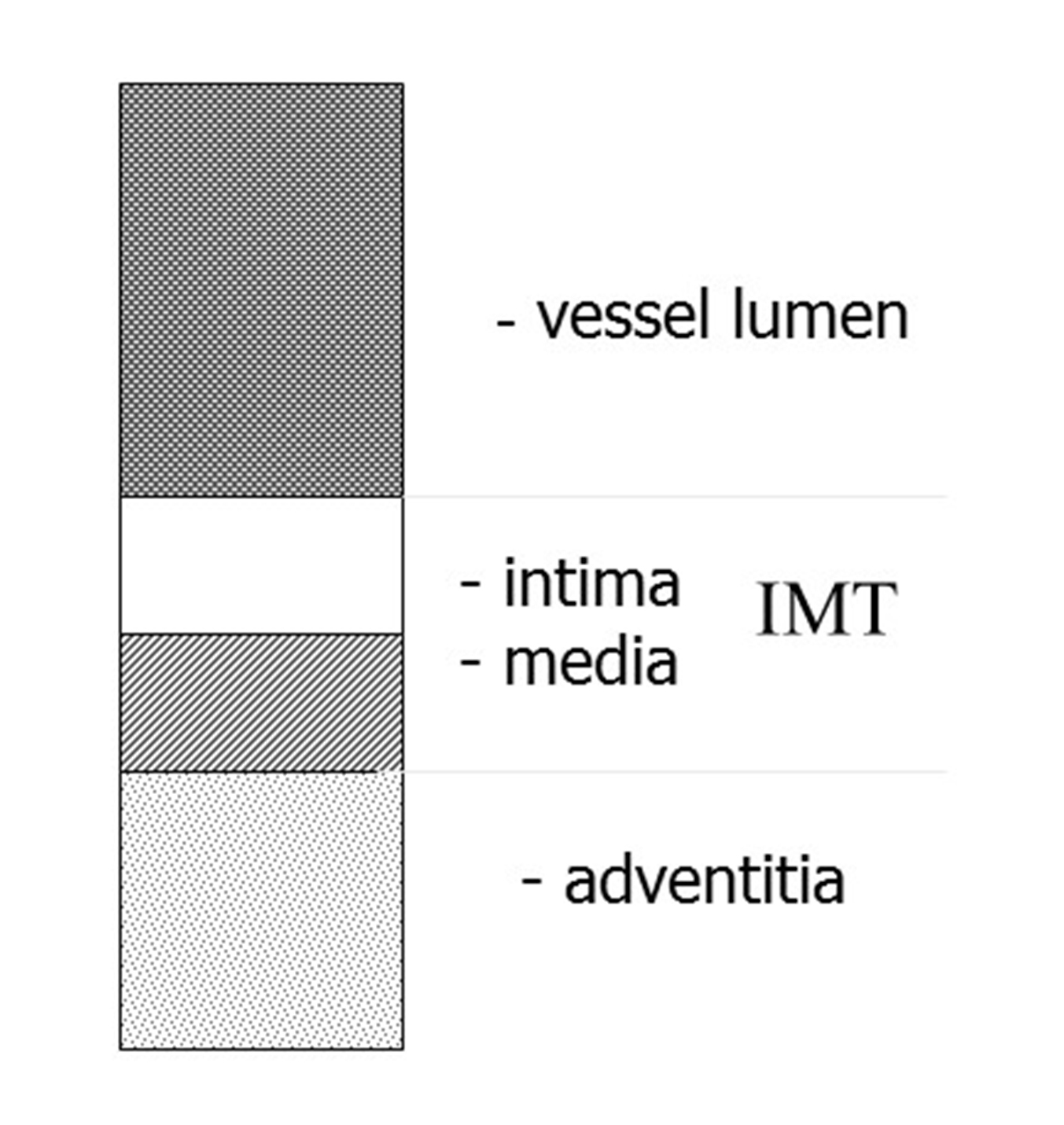

Supplement: Supplementary Figure 1 — The graphic scheme of IMT measurement. [file Image_1.jpg]

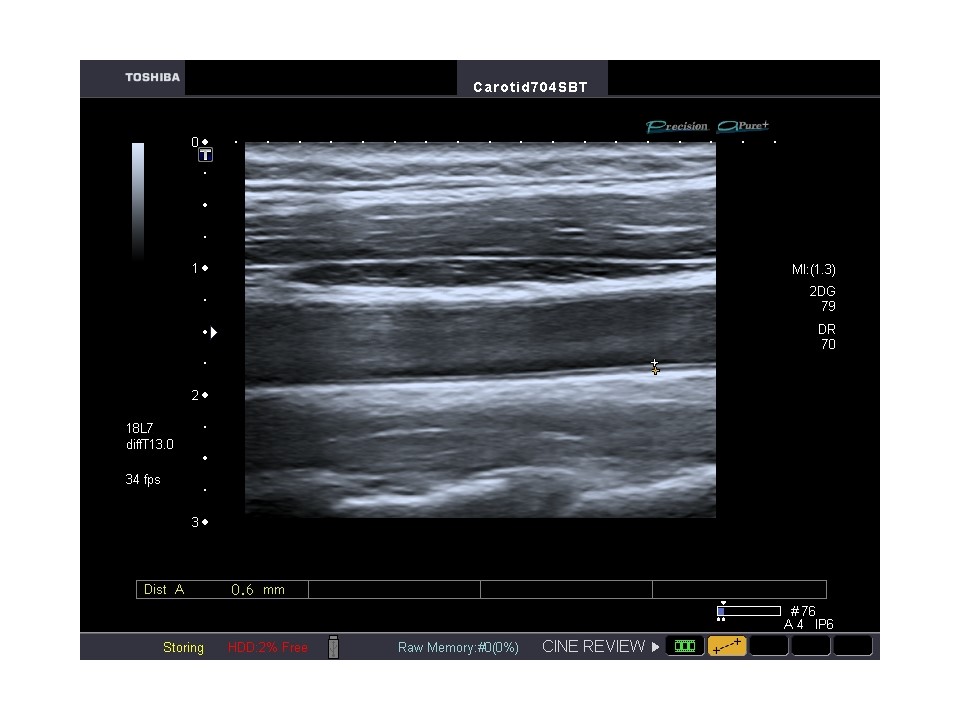

Supplement: Supplementary Figure 2 — IMT ultrasonography representative image for diabetic patient. [file Image_2.jpg]

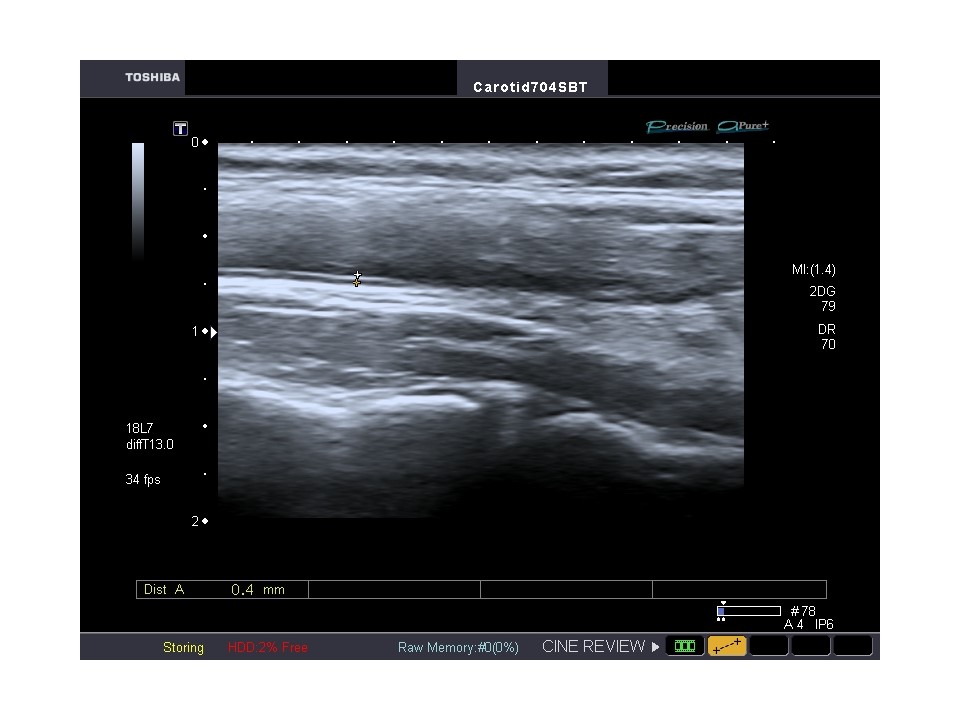

Supplement: Supplementary Figure 3 — IMT ultrasonography representative image for healthy control. [file Image_3.jpg]
